# Supplementary material for: Mutational patterns in chemotherapy resistant muscle-invasive bladder cancer
Source: Nat Commun. 2017 Dec 19;8:2193. doi: 10.1038/s41467-017-02320-7 (PMC5736752; doi:10.1038/s41467-017-02320-7)
Supplement: Supplementary file 1 — Supplementary Information [file 41467_2017_2320_MOESM1_ESM.pdf]

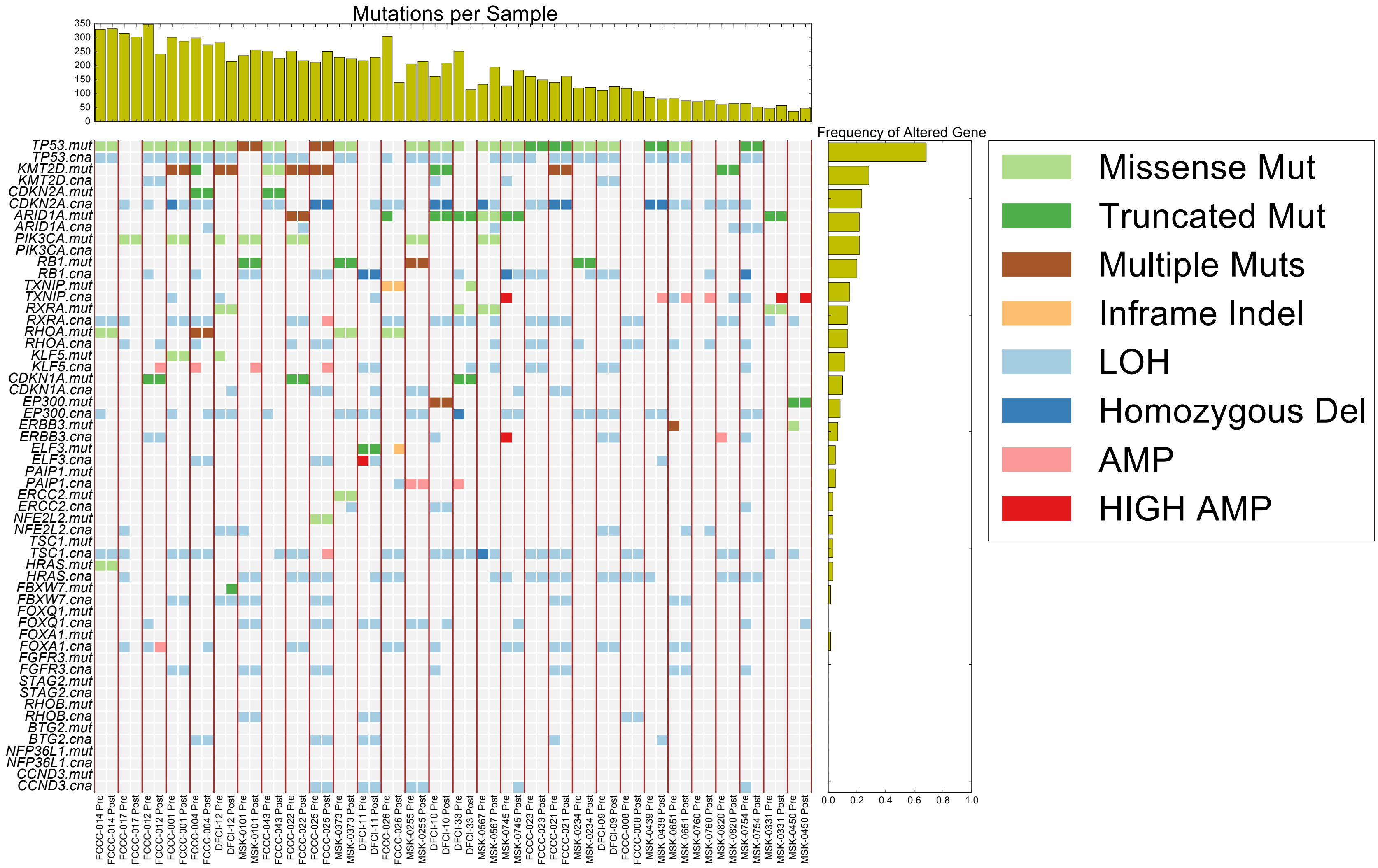

Supplementary Figure 1. Co-Mut plot of matched pre-post samples with mutations and copy number alterations of significantly mutated genes. Red vertical lines separate matched pre-post treatment pairs. The top plot shows mutational load for each tumor, and the altered gene frequency plot on the right shows the frequency of gene alteration within the cohort. A gene alteration is defined here as (1) a nonsynonymous mutation or (2) homozygous deletion or amplification.

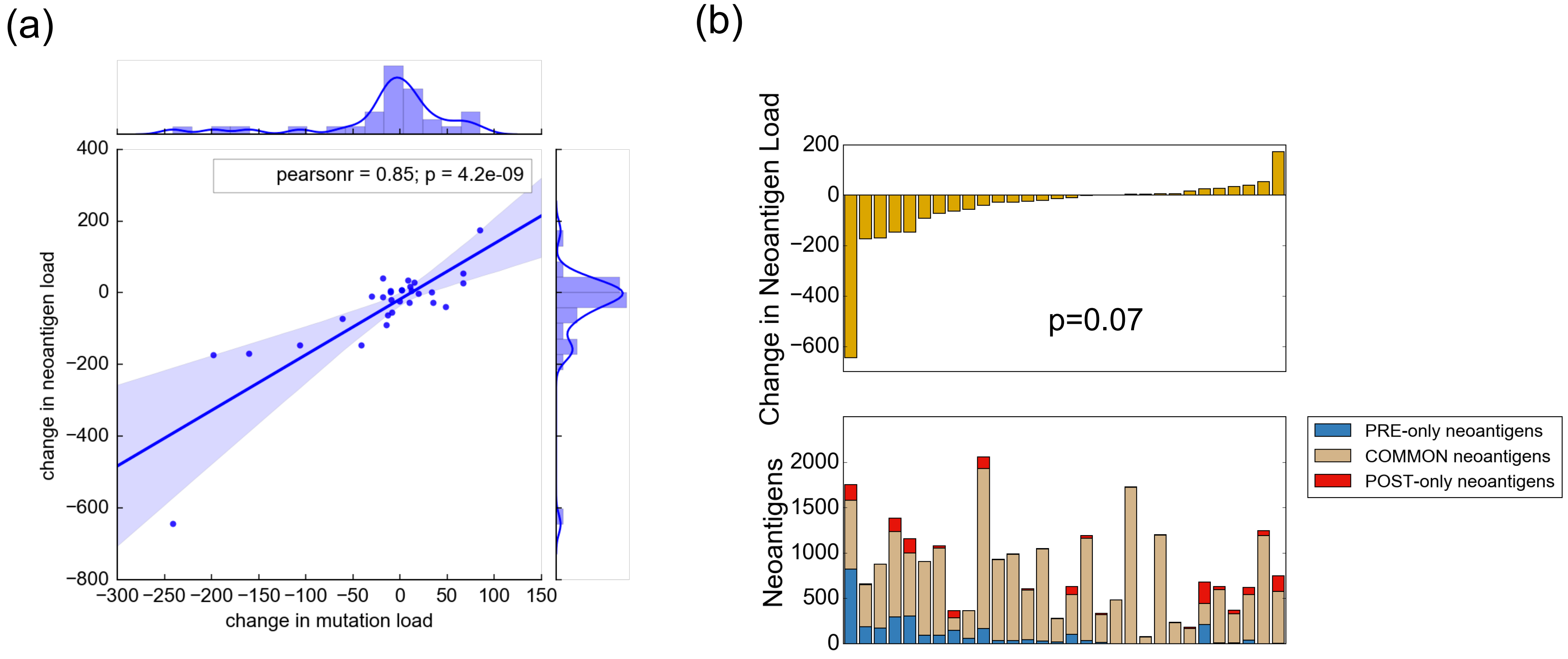

Supplementary Figure 2. Change in Neoantigens between Pre- and Post-Treatment Tumors. (a) Correlation between change in neoantigen vs. mutational burden in pre- and post-treatment burdens. They are highly correlated (Pearson's rho = 0.85, p = 4.2e-09). (b) Change in neoantigen burden. Each column represents one patient. The top graph is the net change in number of neoantigens from matched pre- to post- treatment tumors. The bottom graph represents the breakdown of pre-only, common, and post-only neoantigens in each pair of matched tumors. Overall, there is a trend towards decreased # of neoantigens in the post-treatment tumor (mean change = -41.7, p = 0.07, paired t-test with mean 0).

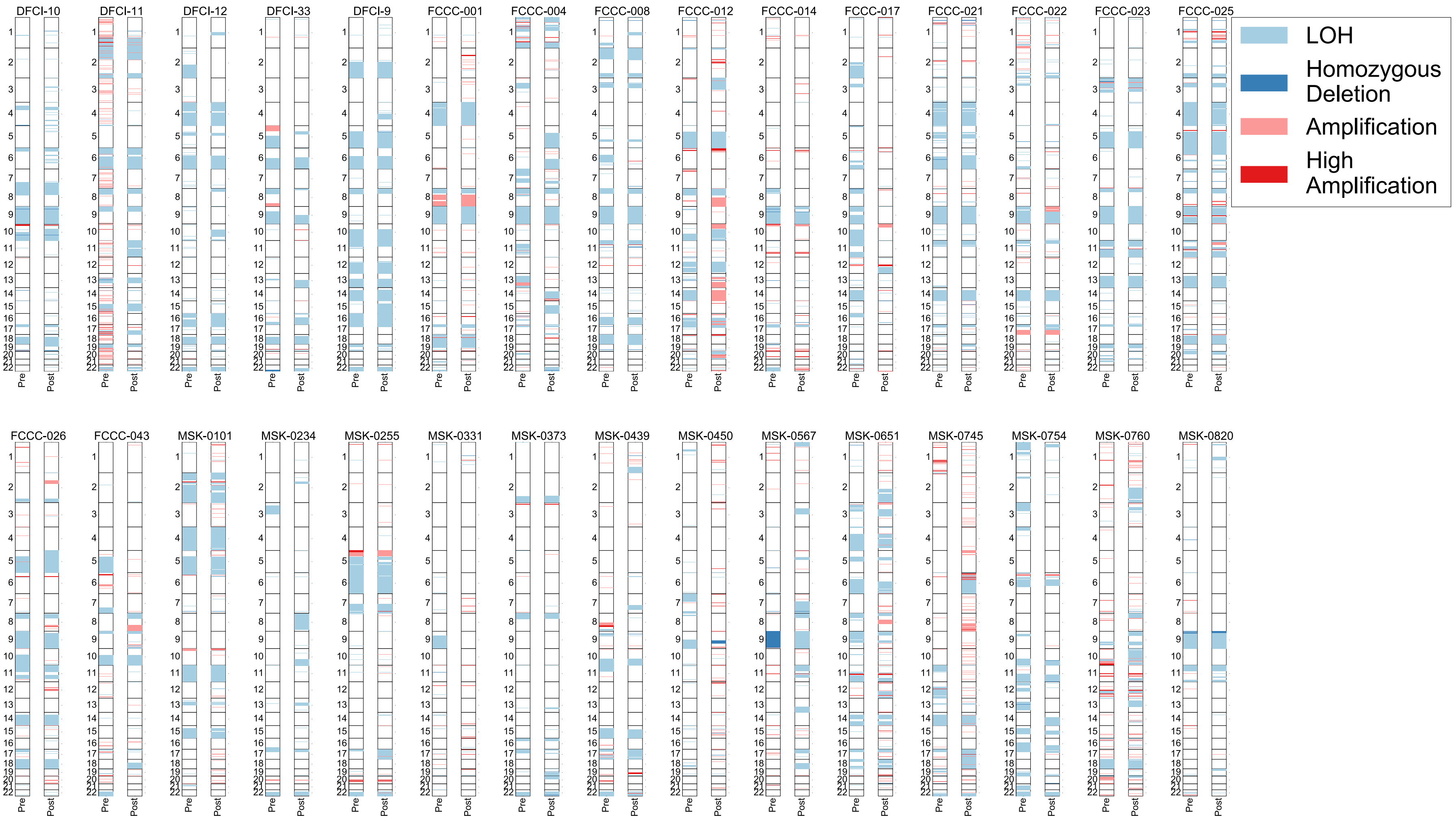

Supplementary Figure 3. Copy number alterations (CNAs) in matched pre- and post- chemotherapy tumor samples.

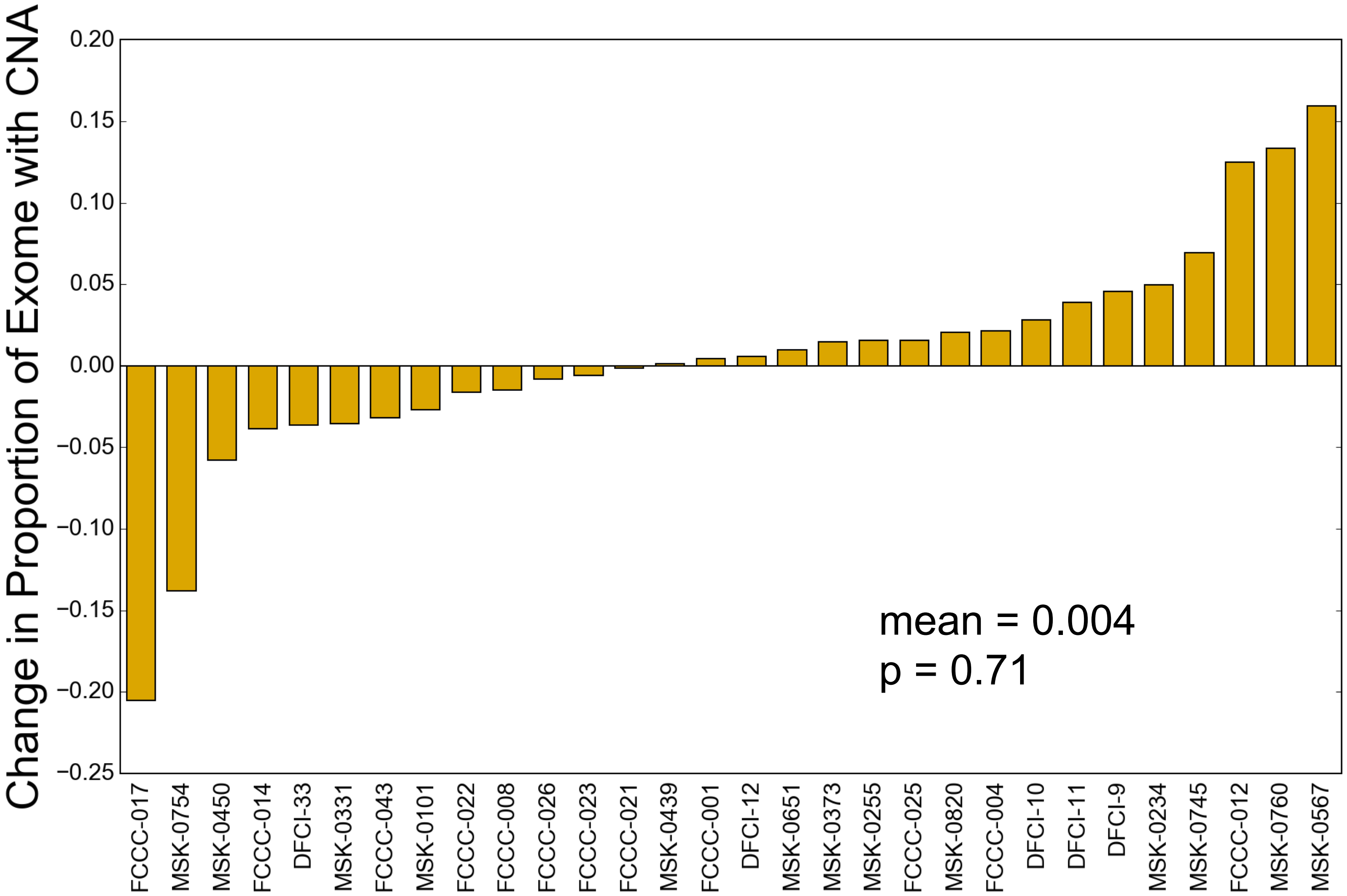

Supplementary Figure 4. Change in Proportion of Exome with CNAs in matched pre- and post-chemotherapy tumor samples**.** Overall, the mean change is 0.4% (p = 0.71, matched t-test with expected mean of 0).

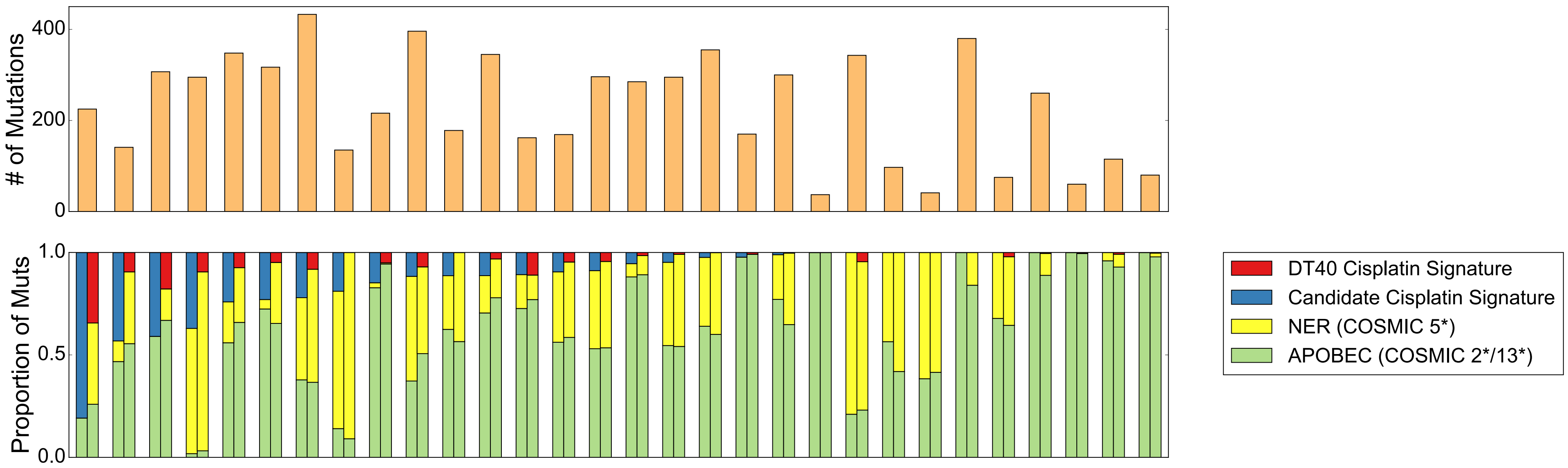

Supplementary Figure 5. Comparison of unknown signature activity and DT40 cisplatin signature activity in post-treatment tumors**.** We inferred the activity level of the DT40 signature by replacing the unknown signature in each post-treatment tumor. Each pair of columns represents a post-treatment tumor, with the proportion of mutations attributed to each signature as stacked bars. The original inferred signature activity is the left column of each pair, and the inferred signature activity replacing the unknown signature with the DT40 cisplatin signature is the right column of each pair. The top graph is the number of mutations per post-treatment tumor.

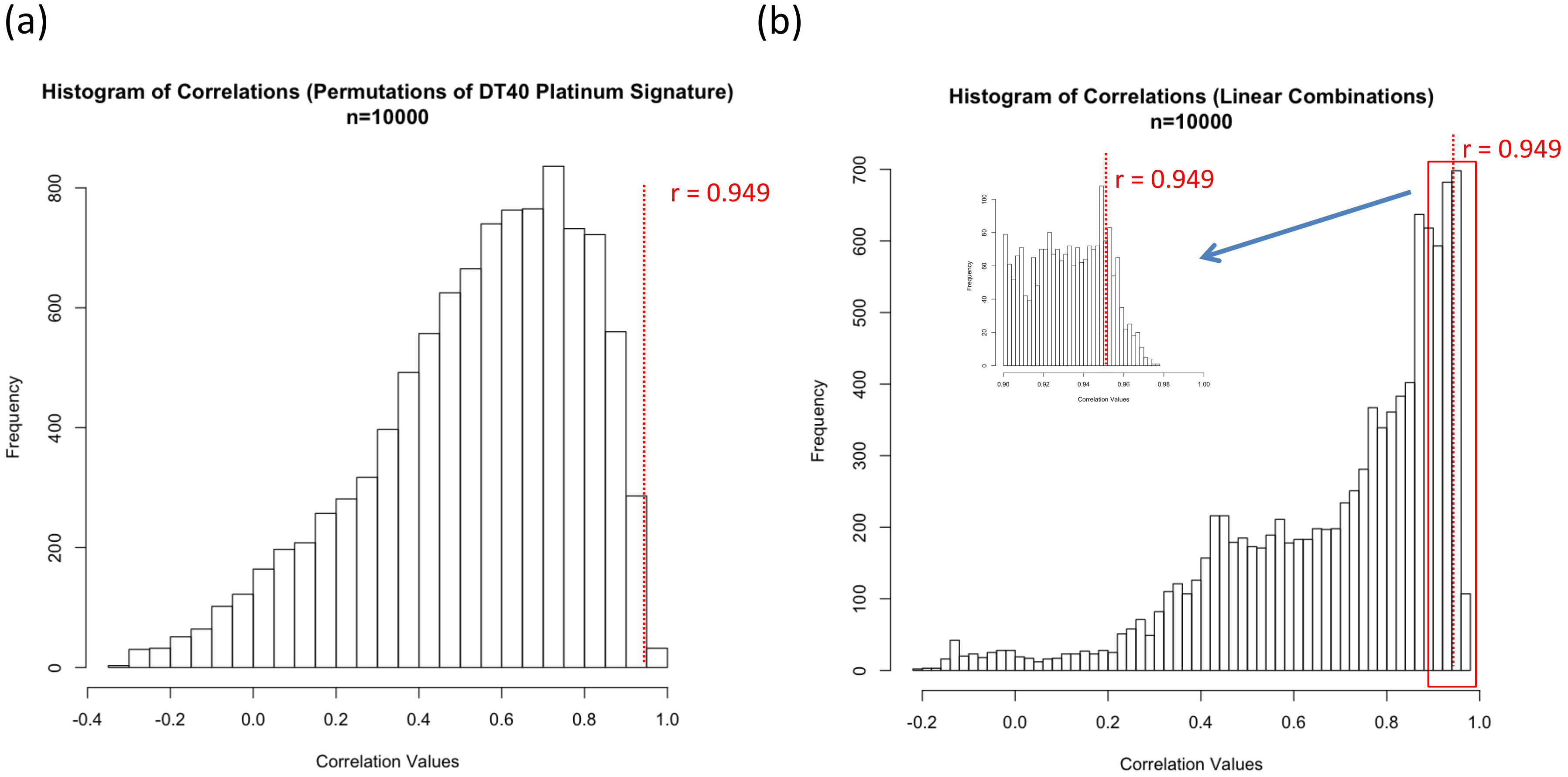

Supplementary Figure 6. Testing Null Distribution of Unknown Signature Replacement

a. Distribution of correlations from replacement of the unknown signature with 10,000 random permutations of DT40 platinum signature. 0.4% of correlations are higher than our discovered correlation (0.949) (empiric p = 0.004).

b. Distribution of correlations from replacement of the unknown signature with 10,000 random linear combinations of 2 COSMIC signatures. 4.9% of correlations are higher than our discovered correlation (empiric p = 0.049). The section with correlation values between 0.9 and 1.0 is magnified inline.

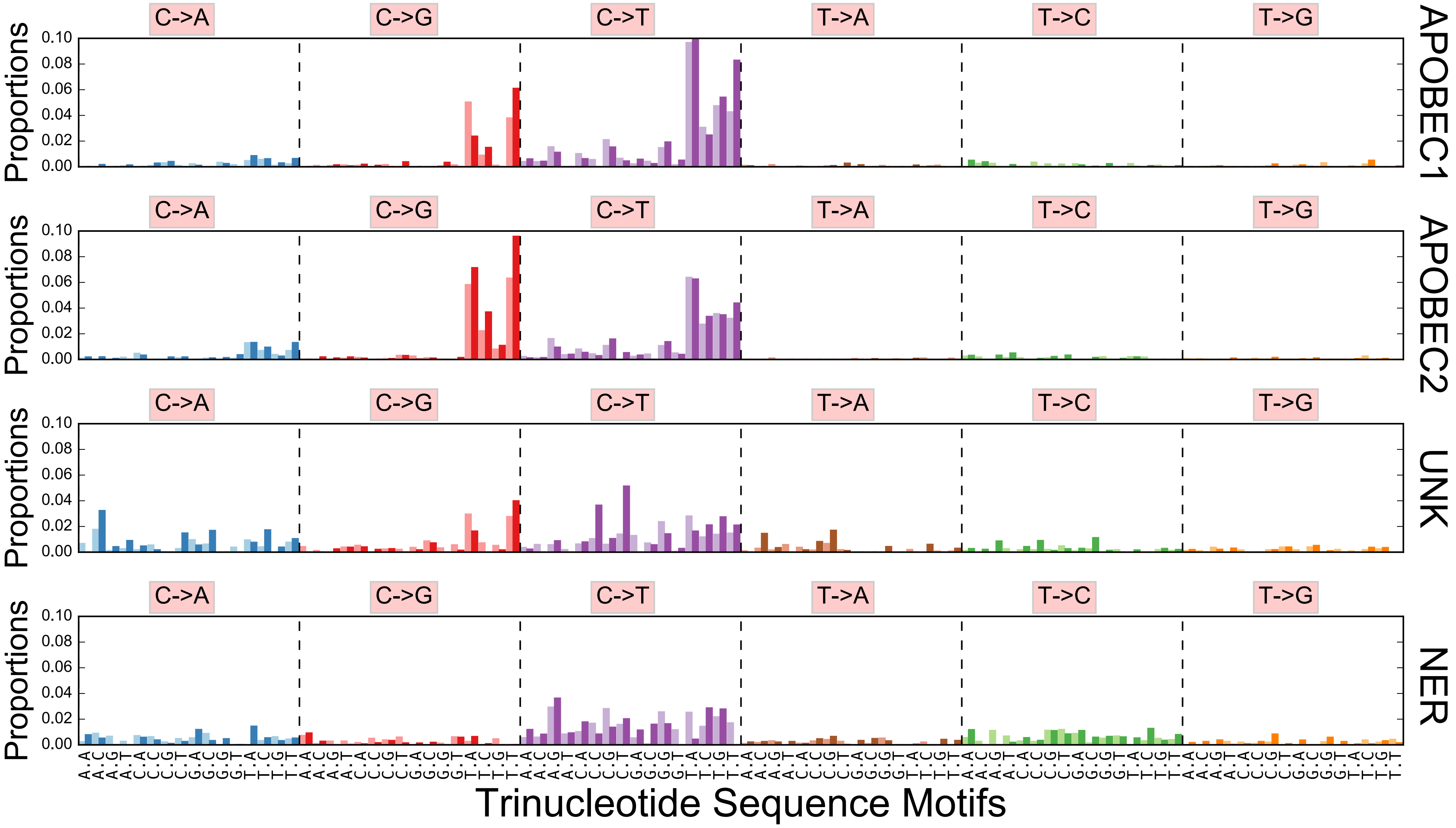

Supplementary Figure 7. Mutational Signature Discovery in Post-Treatment Tumors Accounting for coding (+) and non-coding (-) transcriptional strand SNVs separately. The left (lighter) and right (darker) column for each trinucleotide context correspond to SNVs on coding (+) and non-coding (-) strands, respectively.

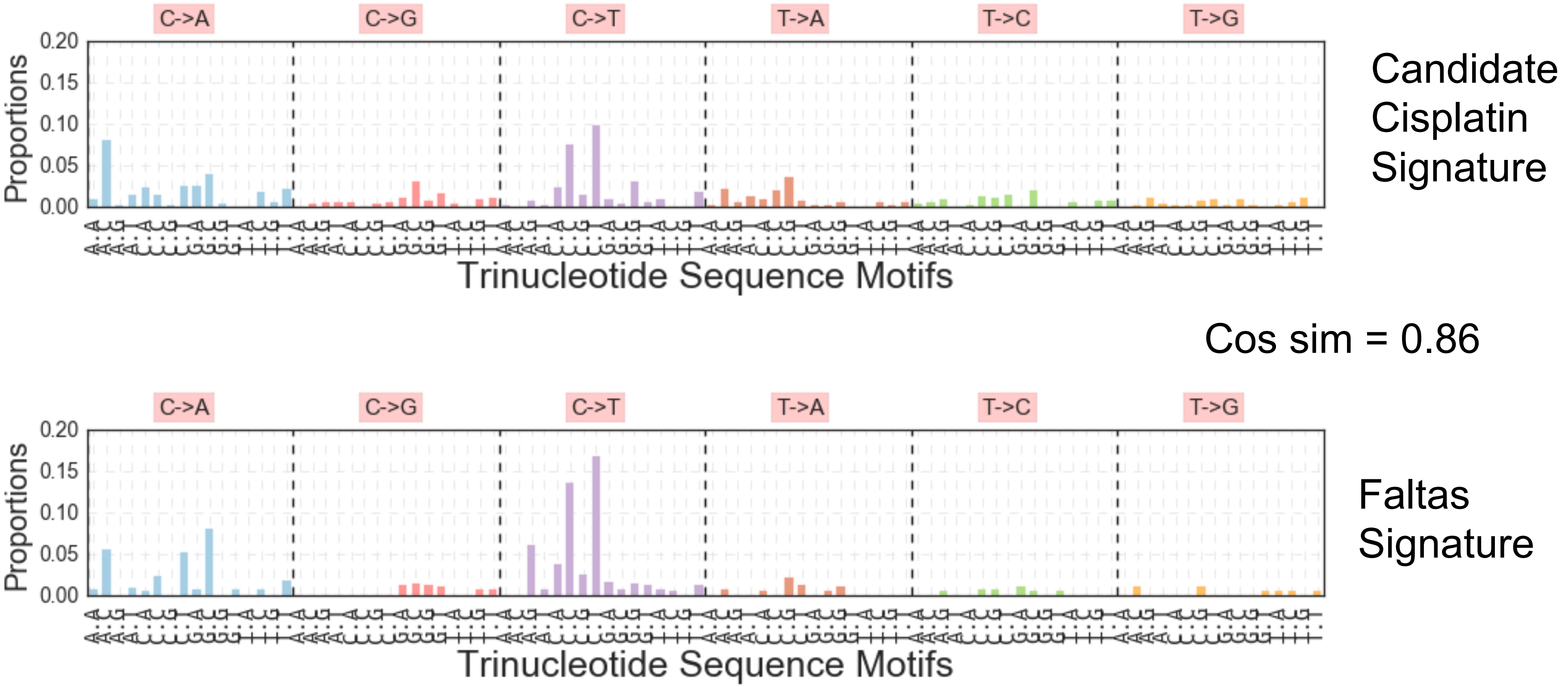

Supplementary Figure 8 Comparison of Mutational Signature in Independent Urothelial Carcinoma Cohort. Our mutational signature discovery process was repeated in an independent cohort of urothelial carcinoma (19 post-platinum-tx urothelial carcinoma tumors with matched pre-tx tumors from 15 pts). A mutational signature (Faltas Signature) was discovered in this validation cohort with good concordance with our candidate cisplatin mutational signature (cos sim = 0.86).

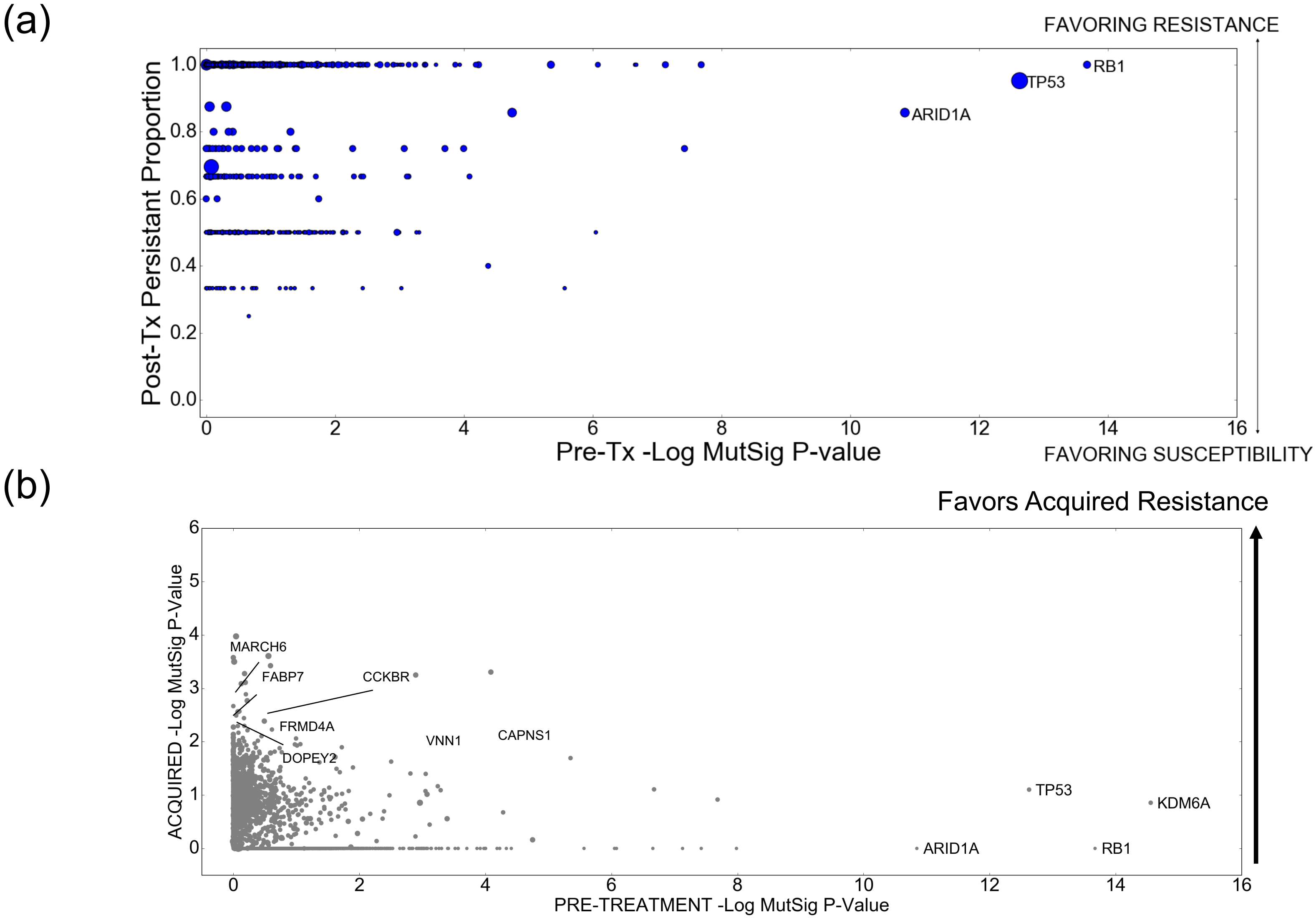

Supplementary Figure 9 Persistent or Acquired Single Gene Mutations in Post-Treatment Samples

(a) Pre-treatment mutations and Persistance in matched post-treatment samples. The y-axis indicates the proportion of mutations in each gene that are detected in both pre and post-treatment samples, and the x-axis indicates the mutational significance of the mutations found in given gene in pre-treatment tumors.

(b) Mutational Significance of "Acquired" (only detected in post-treatment) Mutations vs. their pre-treatment mutations significance. No genes pass FDR (Benjamini-Hochberg) < 0.1.

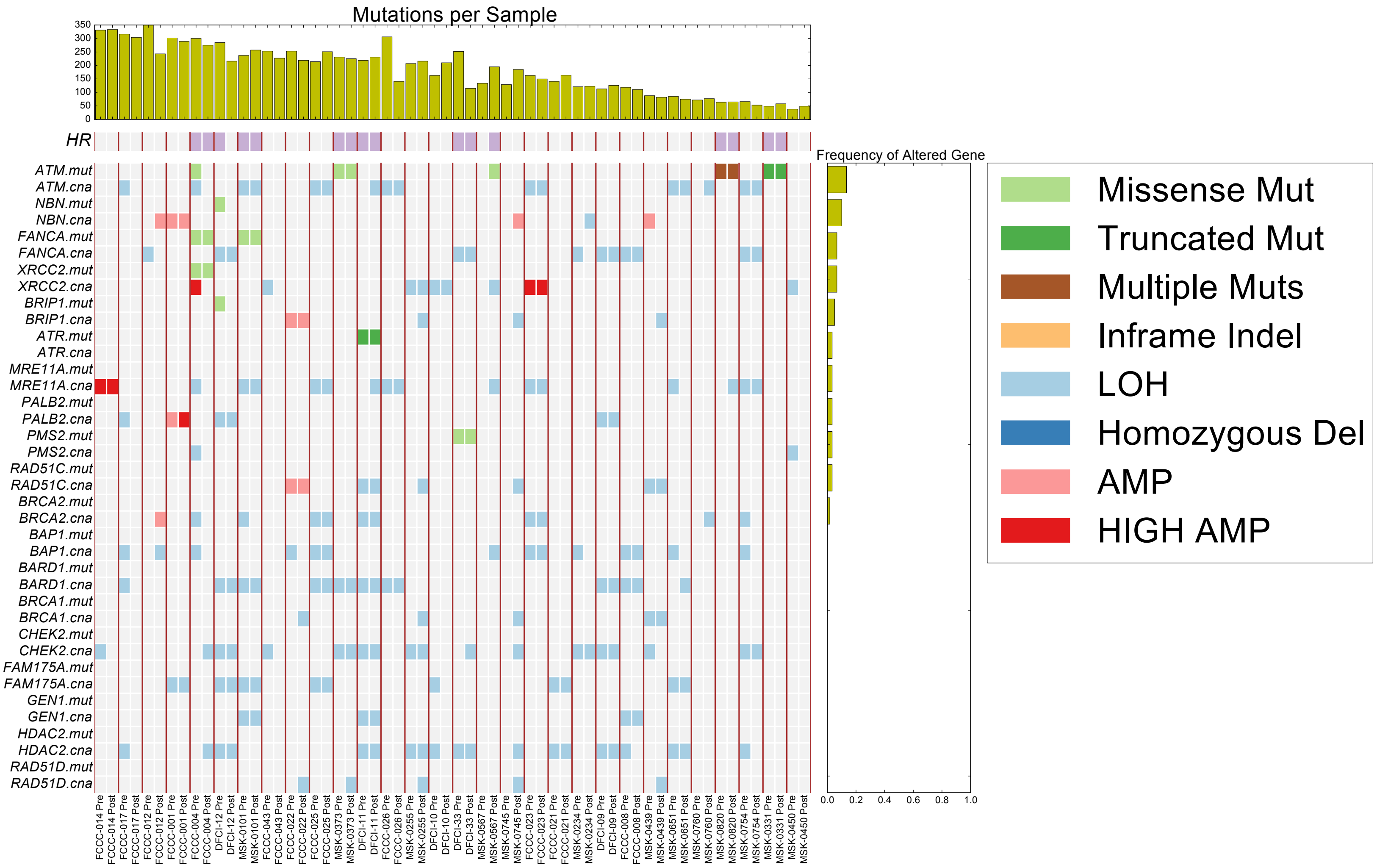

Supplementary Figure 10 Co-Mut plot of matched pre-post samples with mutations and copy number alterations of genes involved in the homologous recombination (HR) DNA damage repair pathways. Red vertical lines separate matched pre-post treatment pairs. The top plot shows mutational load for each tumor, and the altered gene frequency plot on the right shows the frequency of gene alteration within the cohort. A gene alteration is defined here as (1) a nonsynonymous mutation or (2) homozygous deletion or amplification.

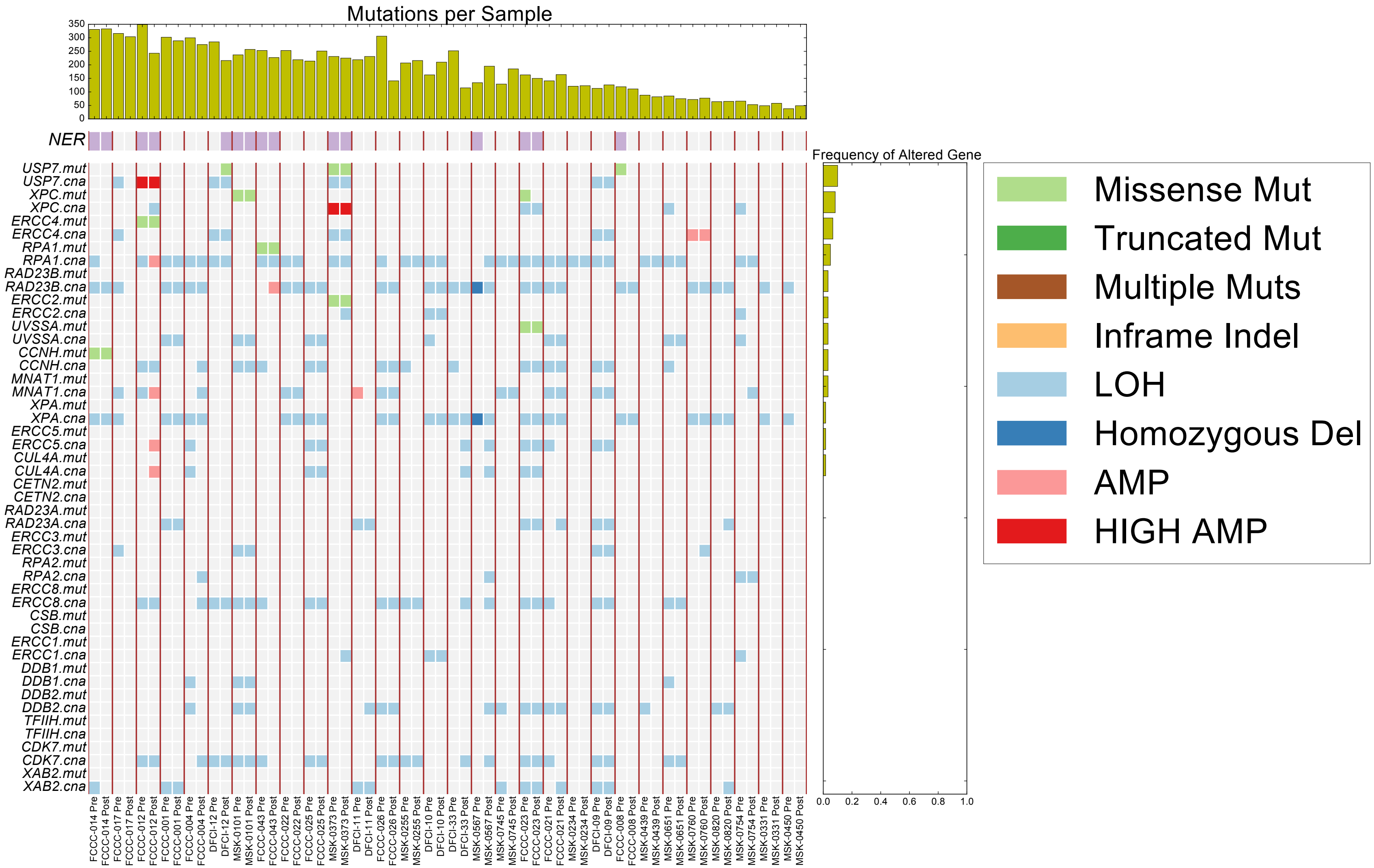

Supplementary Figure 11 Co-Mut plot of matched pre-post samples with mutations and copy number alterations of genes involved in the nucleotide excision repair (NER) DNA damage repair pathways. Red vertical lines separate matched pre-post treatment pairs. The top plot shows mutational load for each tumor, and the altered gene frequency plot on the right shows the frequency of gene alteration within the cohort. A gene alteration is defined here as (1) a nonsynonymous mutation or (2) homozygous deletion or amplification.

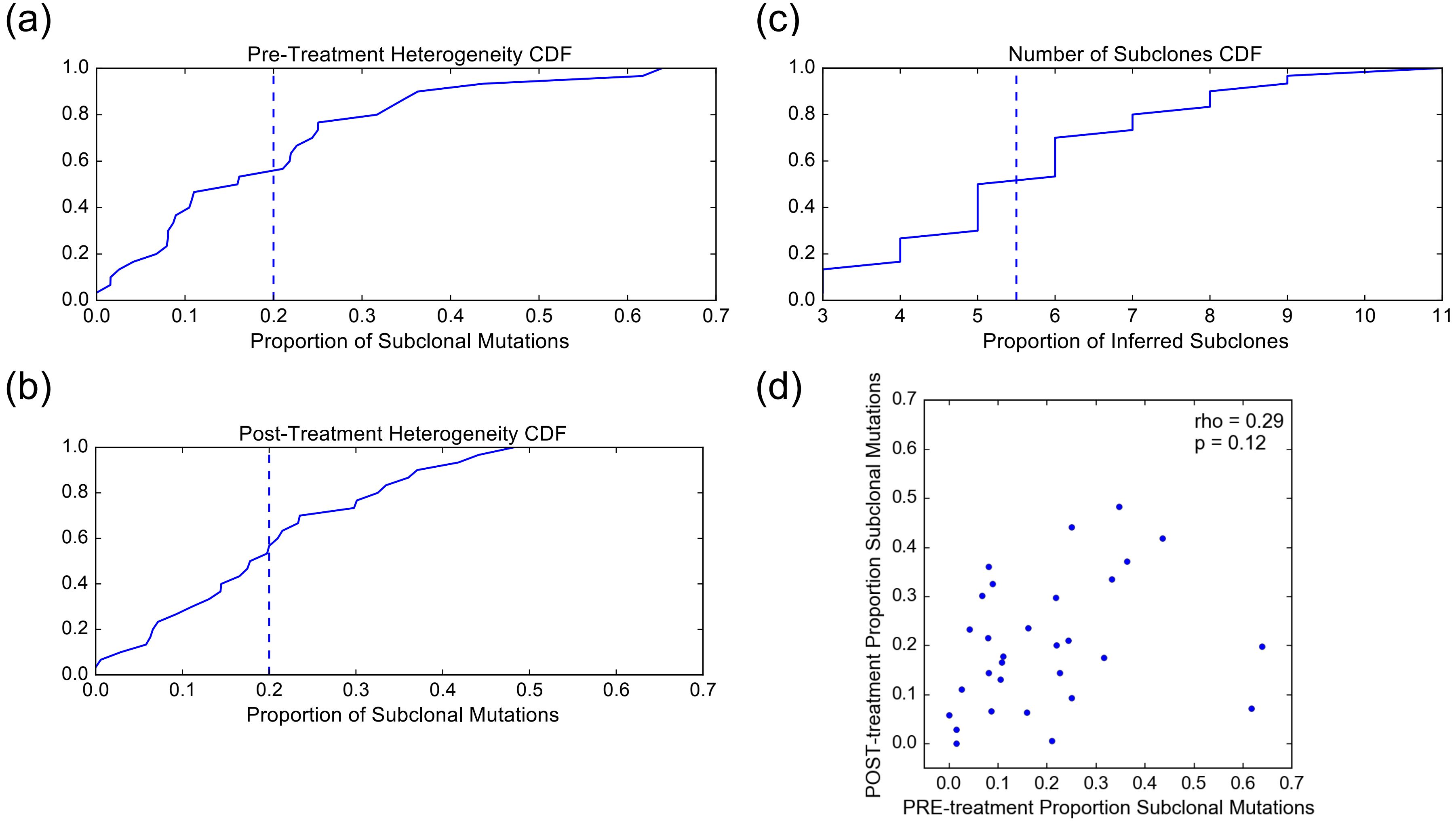

Supplementary Figure 12 Distributions of Measures of Intratumoral Heterogeneity. Heterogeneity is defined as the proportion of mutations that are subclonal or the number of inferred subclones. The dotted line indicates where the cohort was split to define “high” and “low” heterogeneity.

(a) Cumulative Distribution Function (CDF) of Heterogeneity in Pre-Treatment Tumors.

(b) Cumulative Distribution Function (CDF) of Heterogeneity in Post-Treatment Tumors.

(c) Cumulative Distribution Function (CDF) of Number of Inferred Subclones. Heterogeneity is defined as the number of inferred subclones (private to pre- or post-treatment tumors or shared between the two).

(d) Correlation between Pre- and Post-treatment Heterogeneity. There is no statistically significant correlation between pre- and post-treatment heterogeneity (Pearson rho = 0.27, p = 0.15)

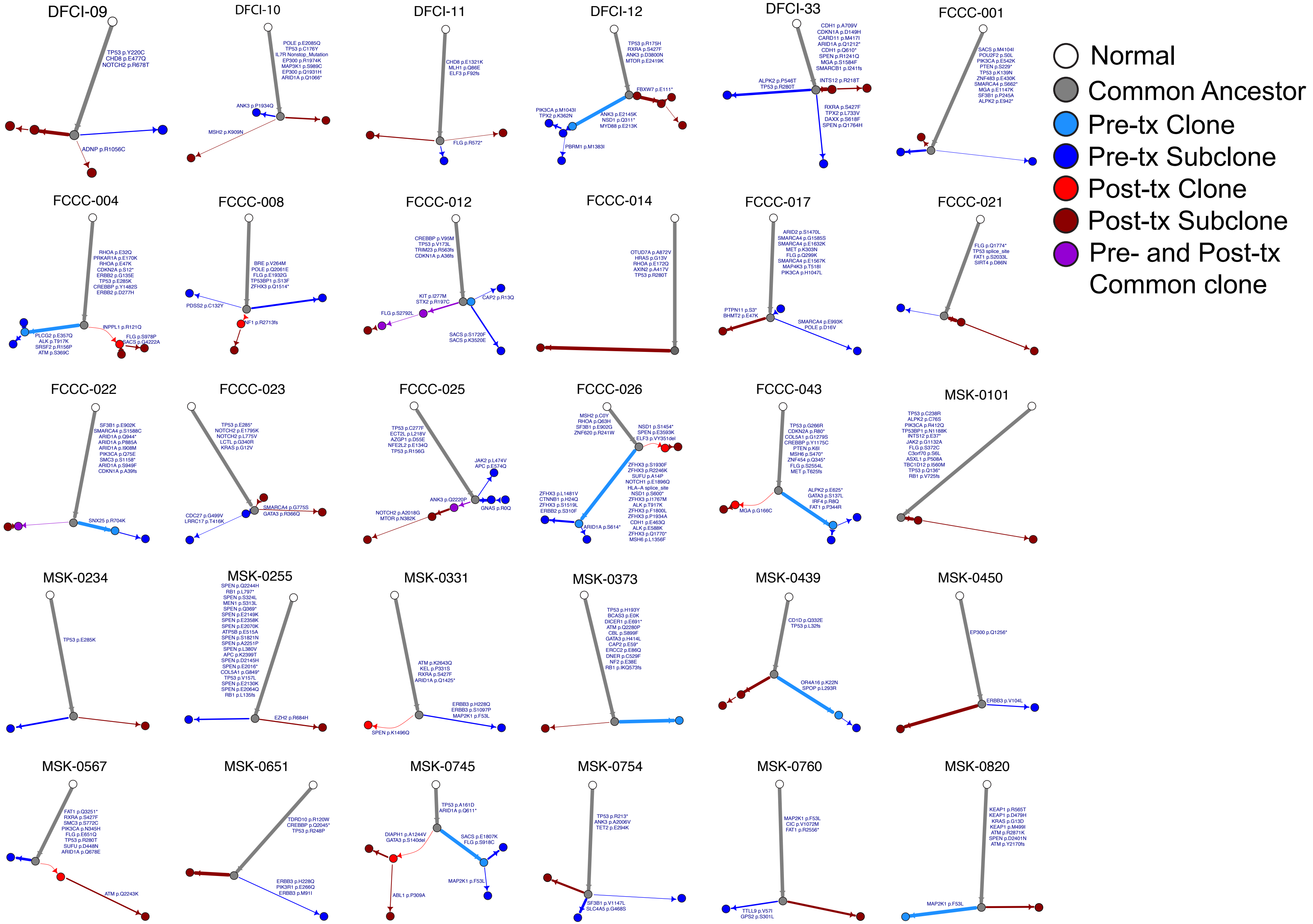

Supplementary Figure 13 Inferred phylogenetic trees for each pair of matched pre- and post-treatment tumors. SNVs and small indels in significantly mutated genes are labeled where they are inferred to have occurred within the phylogenetic tree.

(a)

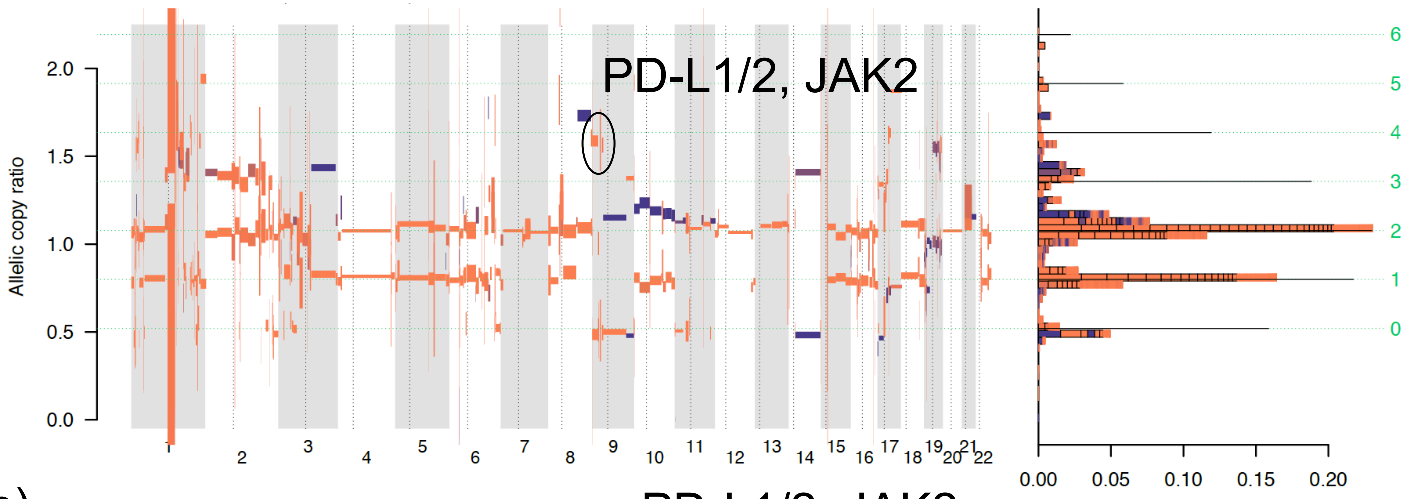

(b)

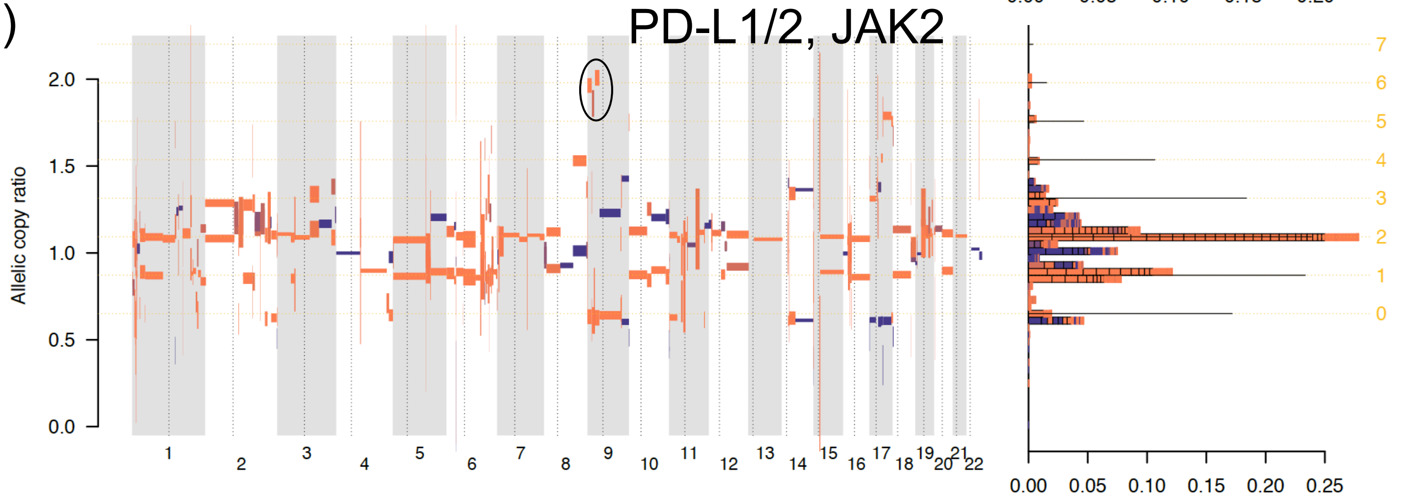

Supplementary Figure 14. Amplification of segment containing PD-L1/2, JAK2 in one patient (FCCC-022)

(a) Inferred Allelic Copy Numbers across the genome of the pre-treatment tumor. The segment containing PD-L1/2, JAK-2 (Chromosome 9p, circled) has an inferred copy number gain (~4) but does not reach the threshold needed to call an amplification.

(b) Inferred allelic copy number across the genome of the post-treatment tumor. The segment containing PD-L1/2, JAK-2 (Chromosome 9p, circled) has a higher inferred copy number (~6) that was called an amplification.

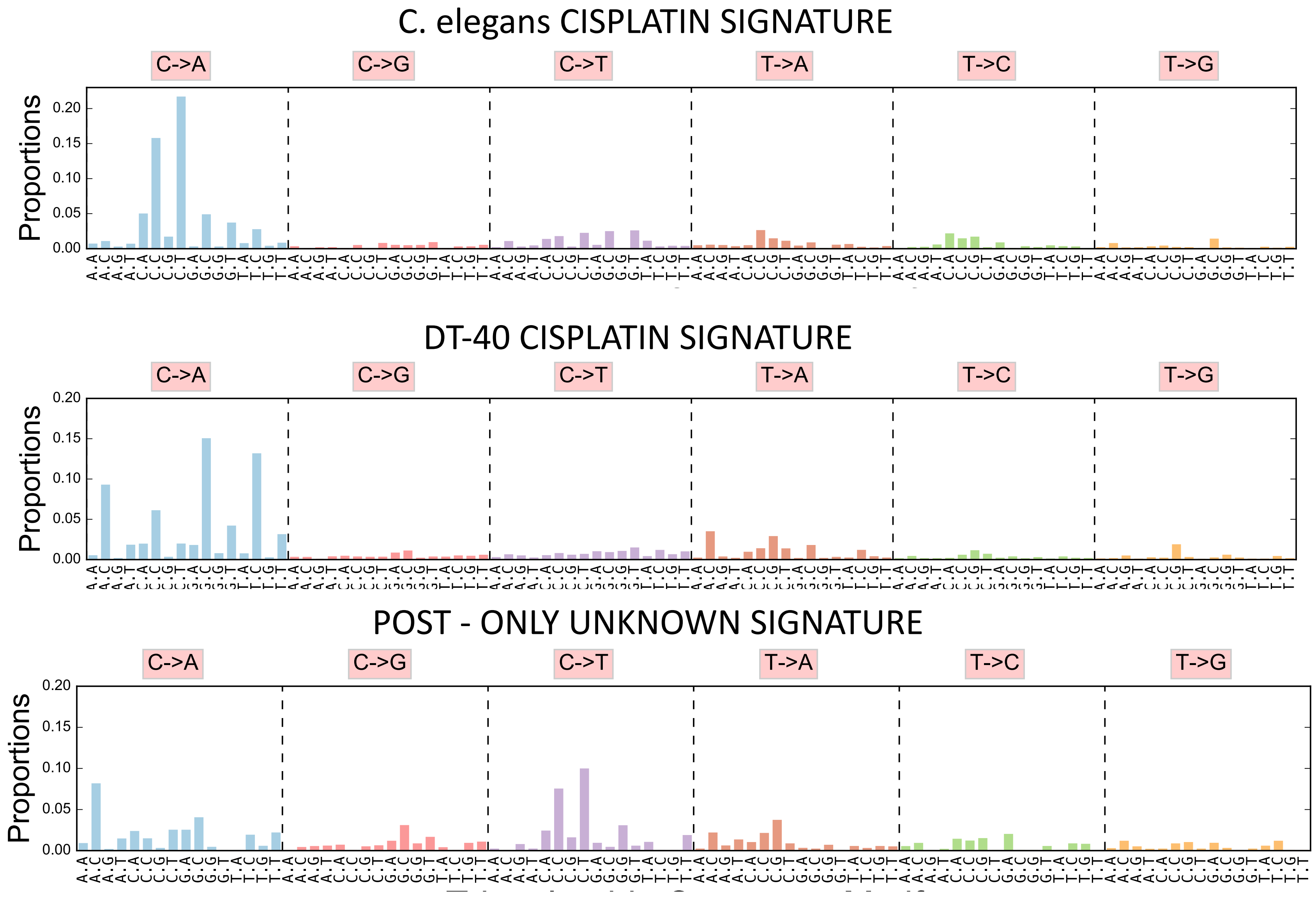

Supplementary Figure 15. Comparison of cisplatin-induced mutational signatures in C. elegans, DT40, and our candidate cisplatin signature. The C. elegans mutational signature is not similar to either the DT40 cisplatin mutational signature (cos sim = 0.44) or our candidate cisplatin signature (cos sim = 0.36)

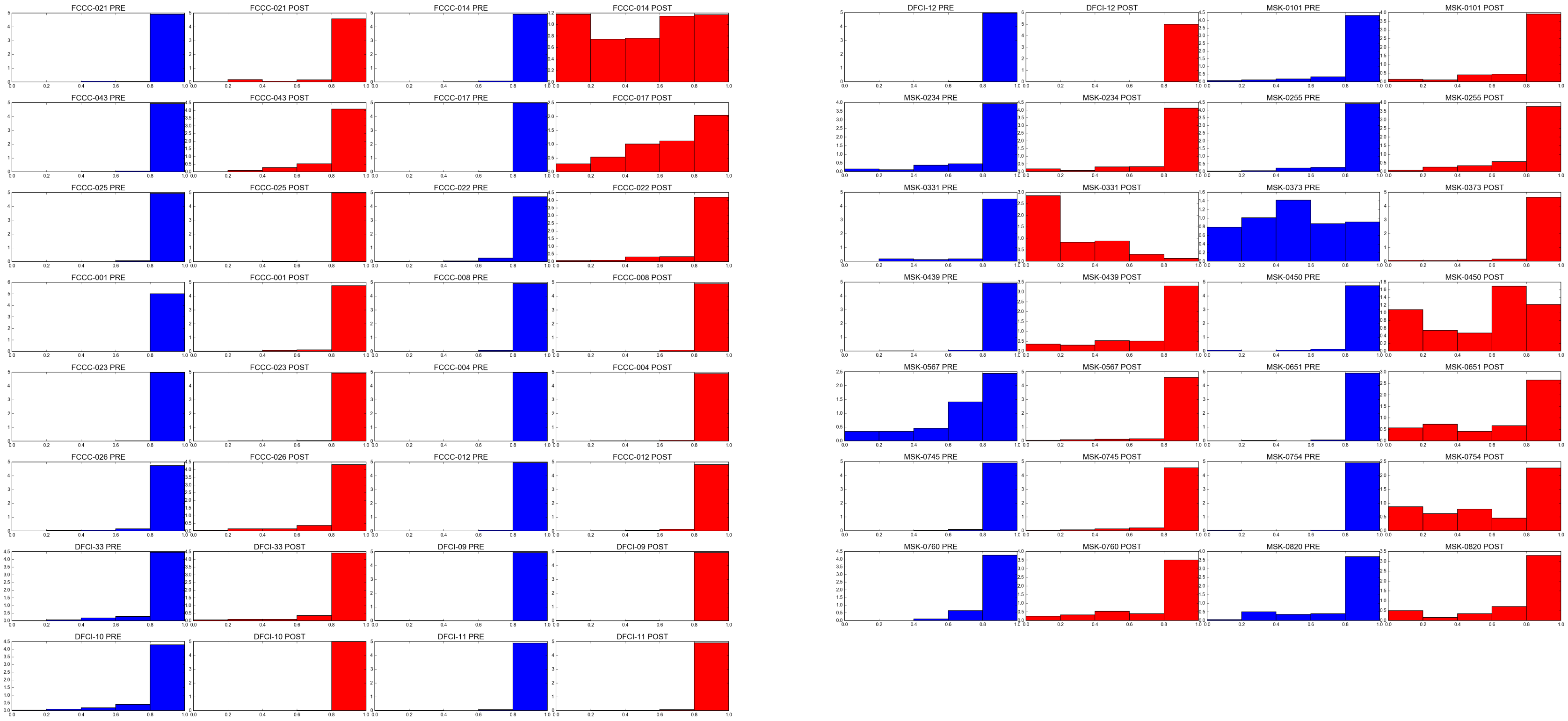

Supplementary Figure 16. Histograms of the power to detect mutations from the union set of mutations in the pre- and post-treatment matched tumors. The x axis shows the power to detect ranging from 0 to 1, and the y axis is a normalized proportion of mutations. Note that the actual set of compared mutations includes those that do not meet the threshold of power to detect (0.8) but are detected in the tumor.

(a)

Variable	HRR (95% CI)	P value
Pre-Treatment Heterogeneity	1.38 (0.81-2.35)	0.24
Age, per 1-yr increase	0.95 (0.48-1.90)	0.9
Gender		
-Male	1.47 (0.77-2.80)	0.24
-Female	1	
pathologic T stage (per increase of 1)	1.44 (0.78-2.67)	0.24
pathologic N stage (per increase of 1)	1.29 (0.71-2.36)	0.4
Treatment		
-MVAC	1	
-GC	0.99 (0.47-2.07)	0.98

(b)

Variable	HRR (95% CI)	P value
Post-Treatment Heterogeneity	1.77 (1.06-2.97)	0.03
Age, per 1-yr increase	1.19 (0.65-2.19)	0.57
Gender		
-Male	1.59 (0.85-2.97)	0.14
-Female	1	
pathologic T stage (per increase of 1)	1.15 (0.59-2.24)	0.68
pathologic N stage (per increase of 1)	1.50 (0.77-2.92)	0.23
Treatment		
-MVAC	1	
-GC	0.82 (0.40-1.69)	0.59

(c)

Variable	HRR (95% CI)	P value
Number of Clones, per increase of one	1.80 (1.13-2.88)	0.014
Age, per 1-yr increase	1.27 (0.68-2.38)	0.45
Gender		
-Male	1.97 (1.00-3.85)	0.049
-Female	1	
pathologic T stage (per increase of 1)	1.27 (0.67-2.39)	0.47
pathologic N stage (per increase of 1)	1.43 (0.79-2.60)	0.24
Treatment		
-MVAC	1	
-GC	0.83 (0.42-1.62)	0.58

Supplementary Table 1. Association of measures of tumor heterogeneity and overall survival, adjusting for clinical covariates. (a) Pre-treatment tumor heterogeneity, measured by the proportion of mutations that are subclonal in the pre-treatment tumor. (b) Post-treatment tumor heterogeneity, measured by the proportion of mutations that are subclonal in the post-treatment tumor. (c) Total number of unique subclones inferred in the pre- and post- treatment tumors.
