# Supplementary material for: Mutational patterns in chemotherapy resistant muscle-invasive bladder cancer
Source: Nat Commun. 2017 Dec 19;8:2193. doi: 10.1038/s41467-017-02320-7 (PMC5736752; doi:10.1038/s41467-017-02320-7)
Supplement: Supplementary file 3 — Description of Additional Supplementary Files [file 41467_2017_2320_MOESM3_ESM.docx]

**Description of Additional Supplementary Files**

File Name: Supplementary Data 1

Description: Key Demographic, Disease, and Survival Characteristics

File Name: Supplementary Data 2

Description: Tumor Sequencing Characteristics

File Name: Supplementary Data 3

Description: Mutations in Matched Pre- and Post-Treatment Tumors. Mutations can be inferred to be “Z” (not-detected), “S” (subclonal), or “C” (clonal) in the pre- and post- treatment tumors, e.g. “S_Z” refers to a mutation that is subclonal in the pre-treatment tumor and not detected in the post-treatment tumor, and “C_C” is a mutation that is clonal in both pre- and post-treatment tumors in the column “var_cluster_classes”.

File Name: Supplementary Data 4

Description: Allelic Gene Copy Number Alterations in Pre- and Post-Treatment Tumors
